# Brain‐Derived Exosomal miR‐9‐5p Induces Ferroptosis in Traumatic Brain Injury‐Induced Acute Lung Injury by Targeting Scd1

**DOI:** 10.1111/cns.70189

**Published:** 2024-12-26

**Authors:** Yi Zhang, Chang Sun, Bailun Wang, Angran Gu, Ziyi Zhou, Changping Gu

**Affiliations:** ^1^ Department of Anesthesiology Shandong Provincial Hospital Affiliated to Shandong First Medical University Jinan Shandong China; ^2^ Department of Anesthesiology, Shandong Provincial Hospital Shandong University Jinan Shandong China; ^3^ School of Anesthesiology Weifang Medical University Weifang Shandong China

**Keywords:** exosomes, Ferroptosis, miR‐9‐5p, Scd1, TBI‐induced ALI

## Abstract

**Aims:**

This study aimed to explore the role and underlying mechanisms of brain‐derived exosomes in traumatic brain injury‐induced acute lung injury (TBI‐induced ALI), with a particular focus on the potential regulation of ferroptosis through miRNAs and Scd1.

**Methods:**

To elucidate TBI‐induced ALI, we used a TBI mouse model. Exosomes were isolated from the brains of these mice and characterized using TEM and NTA. LC–MS analysis revealed an increase in the level of ferroptosis in the lung tissues of mice with TBI. Subsequent miRNA and mRNA sequencing revealed the upregulation of miR‐9‐5p and the downregulation of Scd1 in the pulmonary tissues of these mice. Ferroptosis was assessed by quantifying the levels of ROS, MDA, and Fe^2+^ and the expression of proteins associated with ferroptosis.

**Results:**

TBI led to the release of exosomes enriched with miR‐9‐5p, which targeted Scd1 in lung tissue, thereby promoting ferroptosis. Treatment with antagomir 9‐5p reduced the level of ALI in TBI mice, indicating that exosomal miR‐9‐5p plays a significant role in TBI‐induced ALI.

**Conclusion:**

This study revealed that brain‐derived exosomal miR‐9‐5p mediates ferroptosis in TBI‐induced ALI by targeting Scd1. These findings may provide new insights into the complex interplay between TBI and ALI and highlight the potential of miR‐9‐5p as a target for the development of novel therapeutic strategies.

## Graphical abstract

TBI leads to the release of exosomes enriched with miR‐9‐5p, which targets Scd1 in lung tissue, thereby promoting ferroptosis. Treatment with antagomir 9‐5p alleviates traumatic brain injury‐induced acute lung injury.

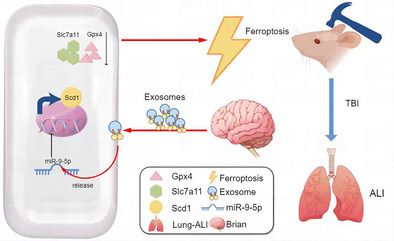

## 1 Introduction

Traumatic brain injury (TBI) is an important cause of disability and death worldwide, causing economic losses of approximately US$400 billion every year [1]. Patients with TBI often face multiple complications, which not only aggravate their condition but also may lead to multiorgan failure, thereby significantly increasing the patient's risk of death [2]. It is imperative to investigate the strong association between TBI and acute lung injury (ALI). Previous studies have indicated that the prevalence of traumatic brain injury‐induced acute lung injury (TBI‐induced ALI) ranges between 20% and 25% and is associated with an increased mortality rate in TBI patients [3]. This association underscores the critical need for a deeper understanding to address the increased risk of pulmonary complications in TBI patients.

Exosomes are vesicles with a diameter of approximately 30–150 nm that are released by cells and are characterized by a typical lipid bilayer membrane structure [4]. Following TBI, almost all brain cells can release exosomes, which possess the remarkable ability to traverse the blood–brain barrier (BBB), thereby gaining access to the systemic circulation and reaching distant organs [5]. Loaded with an array of biological molecules, including DNA, RNA, mRNA, and microRNA (miRNA), exosomes serve as conduits for intercellular communication [6]. miRNAs are a class of noncoding single‐stranded RNAs that negatively regulate gene expression by inhibiting translation or degrading target mRNAs [7]. Recent studies have shown that miRNAs can be transported between disparate cells via exosomes, thereby regulating the functions of other cells [8].

Ferroptosis is a new type of programmed cell death that diverges from apoptosis, necrosis, and autophagy and is triggered predominantly by iron‐mediated lipid peroxidation [9]. Accumulating evidence has underscored the relationship between ferroptosis and conditions such as brain trauma and pulmonary disorders [10, 11]. Ferroptosis also plays an important role in the pathological process of ALI [12, 13]. However, the potential involvement of ferroptosis in the progression of TBI‐induced ALI remains unexplored.

Stearoyl‐CoA (CoA) desaturase‐1 (Scd1) is a lipase located in the endoplasmic reticulum that is responsible for converting saturated fatty acids into monounsaturated fatty acids and is essential for de novo fatty acid synthesis [14]. Scd1 has also been recognized for its significant contributions to immune responses, inflammatory stress, and the regulation of ferroptosis [15]. In cancer research, Scd1 has emerged as a pivotal target in modulating ferroptosis, and its mechanisms of action have been extensively studied [16]. Nevertheless, the regulatory role of Scd1 in ferroptosis in the context of TBI‐induced ALI has not yet been clarified.

This study aimed to explore the role of brain‐derived exosomes in TBI‐induced ALI and the underlying mechanism involved. We hypothesized that brain‐derived exosomes may regulate the ferroptosis process in TBI‐induced ALI by carrying miR‐9‐5p and targeting Scd1.

## 2 Materials and Methods

### 2.1 Exosome Isolation and Identification

Exosomes were isolated through differential centrifugation. The protein concentration of the exosomes was quantified using a BCA protein assay kit (Beyotime, Shanghai, China) according to the manufacturer's protocol. Exosome‐specific markers were detected by Western blotting. The morphology of the exosome samples was determined using transmission electron microscopy (TEM), and the size distribution and concentration of the exosomes were determined using nanoparticle tracking analysis (NTA). The samples were then stored at −80°C for further experimental use.

Brain‐derived exosomes from sham‐operated mice are referred to as “Sham‐Exos,” and those from TBI mice are referred to as “TBI‐Exos.” Exosomes isolated from TBI mice after antagomir NC treatment are termed “TBI‐Exos (antagomir NC),” and those isolated after antagomir 9‐5p treatment are termed “TBI‐Exos (antagomir 9‐5p).”

### 2.2 Animal Experiments

All animal experiments conducted in this study were approved by the Laboratory Animal Ethics Committee of Shandong First Medical University (Approval No. W202406120616). C57BL/6 wild‐type mice (12 weeks old, weighing 16–20 g) were obtained from Vital River Laboratories (Beijing, China) and maintained in a stable environment with controlled temperature and humidity (22°C ± 2°C, 50% ± 10%) and a 12‐h light/dark cycle, with free access to water and food.

### 2.3 Mouse Models of TBI

The animal model was established based on previous studies [17]. Mice in the TBI group were anesthetized with isoflurane in a gas mixture containing 70% N_2_O and 30% O_2_. A bone flap (3.5 mm) was removed at the intersection of the right lower frontal fontanel and the coronal suture, and a controlled cortical impact (CCI) device was used to inflict brain trauma with specific parameters: a tip diameter of 3.5 mm, an impact velocity of 6 m/s, and an impact depth of 2 mm. After completion of the procedure, the scalp was sutured, and the mice were allowed to recover on a warm heating pad. The mice in the sham surgery group underwent craniotomy without CCI. 24 h after surgery, the mice were euthanized by intraperitoneal injection of an overdose of anesthetic, and lung tissue, brain tissue, plasma, and bronchoalveolar lavage fluid (BALF) were collected for subsequent experiments.

### 2.4 Analysis of Cells and Inflammatory Cytokines in Bronchoalveolar Lavage Fluid (BALF)

BALF was collected as previously described [18]. Briefly, the left bronchial tubes of the mice were ligated, and the ice‐cold PBS (0.5 mL) was instilled twice into the right lungs. Then, the collected BALF was centrifuged to pellet the cells and the supernatant was kept at −80°C until it was used for cytokine analysis.

### 2.5 In Vivo Exosome Injection and Drug Treatment

To investigate the effects of brain‐derived exosomes, Sham‐Exos/TBI‐Exos/TBI‐Exos (antagomir NC)/TBI‐Exos (antagomir 9‐5p) (100 μg) were injected into the mice via the tail vein, with an equal volume of PBS serving as a negative control [19]. The mice were sacrificed 24 h later to assess pulmonary inflammation and injury.

GW4869, an exosome secretion inhibitor purchased from Selleck Chemical, was administered to mice via intraperitoneal injection at a dosage of 1.25 mg/kg following TBI, with an equal volume of 0.9% NaCl serving as a control [20].

Ferrostatin‐1 (Fer‐1), an iron death inhibitor also obtained from Selleck Chemical, was administered via tail vein injection at a dosage of 0.8 mg/kg following TBI, with an equal volume of 0.9% NaCl serving as a control [21].

### 2.6 In Vivo Antagomir 9‐5p Treatment

Animal intracerebroventricular injections were performed according to previous study protocols [22]. Under general anesthesia, antagomir NC or antagomir 9‐5p (GenCefe Biotech Co. Ltd., Jiangsu, China) was injected into the right lateral ventricle of the brain using a stereotactic apparatus. The injection concentration was 2 μM, with the following coordinates (relative to the bregma): anteroposterior +1.5 mm, mediolateral +1 mm, and dorsoventral −2 mm. Injections were administered 15 min after CCI.

To study the therapeutic effect of antagomir 9‐5p, intranasal administration was performed according to the manufacturer's instructions and previous methods [23]. A total of 40 nmol of antagomir 9‐5p was dissolved in 1 mL of RNase‐free water and administered in 24 μL aliquots (1 nmol per mouse) via the nostrils using a pipette, alternating between the left and right nostrils, with an interval of 3–5 min between applications. The mice showed no signs of respiratory obstruction or other abnormalities, and all survived the procedure.

### 2.7 Hematoxylin–Eosin (HE) Staining and Lung Injury Scoring

To evaluate the morphological changes in the lung tissue, the lung specimens were fixed in 4% formalin, paraffin‐embedded, and then sectioned into 5 μm slices. These sections were subsequently stained with HE and examined under a light microscope (Nikon, Tokyo, Japan). To determine the severity of lung injury in mice, a scoring system from 0 to 4 was used to classify the degree of lung injury [24].

### 2.8 Wet/Dry (W/D) Weight Ratio

The W/D weight ratio was used to assess the severity of pulmonary edema. The right lung tissue of each mouse was extracted, and any surface moisture was carefully removed with gauze. The wet weight of the lung was then measured. The lung tissue was subsequently dried in an oven at 60°C for 48 h and reweighed to determine the dry weight. Finally, the W/D weight ratio was calculated.

### 2.9 Enzyme‐Linked Immunosorbent Assay (ELISA)

The levels of IL‐1β, IL‐6, and TNF‐α in the BALF were assessed using ELISA kits (Reed Biotech, Wuhan, China) according to the manufacturer's instructions.

### 2.10 Cell Culture and Transfection

Mouse lung epithelial cells (MLE‐12) were obtained from the FuHeng Cell Center (Shanghai, China). The cells were cultured in DMEM/F‐12 supplemented with 1% streptomycin/penicillin solution and 10% fetal bovine serum (Lonsera, Shanghai, China).

MLE‐12 cells were cocultured with exosomes isolated from TBI and sham mice (20 μg/mL) for 24 h, followed by transfection. The plasmids were transfected into cells using Lipofectamine 3000 (Invitrogen, Carlsbad, CA, USA) according to the manufacturer's instructions. The miR‐9‐5p mimics and miR‐9‐5p inhibitor were constructed by GenePharma (Shanghai, China), and the overexpression plasmid for Scd1 was constructed by Keyybio (Jinan, Shandong, China). The sequences of the miR‐9‐5p mimics and the miR‐9‐5p inhibitor are as follows:

mmu‐miR‐9‐5p mimics sequence: 5‐UCUUUGGUUAUCUAGCUGUAUGA‐3.

mmu‐miR‐9‐5p mimics antisense sequence: 5‐AUACAGCUAGAUAACCAAAGAUU‐3.

mmu‐miR‐9‐5p inhibitor sequence: 5‐UCAUACAGCUAGAUAACCAAAGA‐3.

### 2.11 Exosome Uptake Assay

Neutrophil‐derived exosomes were tagged with PKH67 green fluorescence cell linker mini kit (Sigma MINI67‐1KT) in light of the manufacturer's instructions. Briefly, 1.5 mL EP was prepared for controlled exosome dilution. Exosomes were resuspended with 500 μL of Diluent C solution, and 2 μL of PKH67 was added to 500 μL Diluent C to prepare PKH67 Diluent. Next, the prepared exosome diluent and PKH67 diluent were mixed well and incubated at 37°C for 5 min. Following incubation, 8 mL of 15% complete exosome‐free medium was added to terminate the staining reaction. The exosomes were precipitated with an equal volume of PEG overnight at 4°C and centrifuged at 10,000 ***g*** for 1 h at 4°C. The exosomes were resuspended in PBS (pH = 7.4) and stored at −80°C. On the second day, exosomes were added and incubated with cells for 24 h, then washed with PBS for three times. The cytoplasm was stained with fibrillarin (F‐actin). Cells were fixed with 4% paraformaldehyde (PFA) for 10 min at room temperature, and permeabilized with 0.5% Triton X‐100 in PBS for 5 min. Nuclei were stained by 4′,6‐diamidino‐2‐phenylindole (DAPI) for 10 min. Fluorescence was observed under a confocal microscopy.

### 2.12 Determination of Ferrous Ion (Fe^2+^), Reactive Oxygen Species (ROS), Malondialdehyde (MDA), and Glutathione (GSH)

MLE‐12 cells were treated with a ROS assay kit (Beyotime, Shanghai, China) and a ferrous iron detection probe (Dojindo Molecular Technologies Inc.) according to the manufacturer's instructions, and the fluorescence intensity was measured under a fluorescence microscope.

The concentrations of Fe^2+^ and MDA, as well as the GSH/GSSG ratio in MLE‐12 cells and lung tissue, were analyzed using Fe^2+^ detection kits (Solarbio, Beijing, China), MDA detection kits (Beyotime, Shanghai, China), and GSH detection kits (Beyotime, Shanghai, China).

### 2.13 CCK8 Assay

The CCK8 assay was performed according to the manufacturer's instructions (Beyotime, Shanghai, China). A total of 2000 cells (in 100 μL of culture media) were added to each well of a 96‐well plate and cultured for 24, 48, or 72 h. At each time point, 10 μL of sterile CCK‐8 was added to each well, and the plate was incubated for an additional 2 h at 37°C. The absorbance at 450 nm was measured using a microplate reader.

### 2.14 miRNA Sequencing

Lung tissues were obtained from sham‐operated mice and TBI model mice and sent to Genesky Biotechnologies Inc. (Shanghai, China) for miRNA sequencing. Total RNA was extracted from the lung tissues, and its concentration was determined, followed by 2 × 150 sequencing on the Illumina MiSeq platform. The threshold for differentially expressed miRNAs (DE‐miRNAs) was set to an absolute log2Fold Change (FC) > 1, with a *p* value after data preprocessing of less than 0.05.

### 2.15 mRNA Sequencing

Lung tissues were harvested from both the sham and TBI groups of mice. Total RNA was subsequently extracted from the lung tissues using TRIzol reagent (Invitrogen, USA) and assessed for quality by OBiO Technology (Shanghai, China) with a NanoDrop 2000 spectrophotometer (Thermo Scientific, USA) and an Agilent 2100 bioanalyzer (Agilent Technologies, USA). Qualified libraries were sequenced on the Illumina NovaSeq 6000 platform using the PE150 methodology. Significantly differentially expressed genes were filtered based on a padj < 0.05 and an absolute fold change (|FC|) ≥ 2.

### 2.16 LC‐MS Analysis

The metabolomic data analysis was performed by Shanghai Luming Biological Technology Co. Ltd. (Shanghai, China). An ACQUITY UPLC I‐Class plus (Waters Corporation, Milford, USA) fitted with a Q Exactive mass spectrometer equipped with a heated electrospray ionization (ESI) source (Thermo Fisher Scientific, Waltham, MA, USA) was used to analyze the metabolic profile in both ESI‐positive and ESI‐negative ion modes. The original LC‐MS data were processed with Progenesis QI V2.3 software (Nonlinear, Dynamics, Newcastle, UK) for baseline filtering, peak identification, integrals, retention time correction, peak alignment, and normalization.

### 2.17 Luciferase Assay

To explore the targeting of SCD1 by miR‐9‐5p, HEK 293 T cells were co‐transfected with luciferase vectors containing wild‐type (WT) or mutant (MUT) 3′‐UTR of SCD1 and miR‐9‐5p mimics or NC mimics using lipofectamine 3000 reagent (Invitrogen, Carlsbad, CA, USA). After 6 h, the medium was replaced with a complete medium. At 48 h after transfection, the luciferase activities were detected with a dual‐luciferase kit (Beyotime, Shanghai, China). Dual‐luciferase ratios were calculated, and then, the ratio differences among different groups were compared.

### 2.18 Western Blot

After in vivo and in vitro experimental treatments, proteins were extracted from lung tissues and MLE‐12 cells and lysed with RIPA lysis buffer containing protease and phosphatase inhibitors (YaEnzyme, Shanghai, China). Protein concentrations were determined using a BCA protein assay kit (Beyotime, Shanghai, China). Equal amounts of protein samples were separated on a 10% polyacrylamide gel by SDS‐PAGE and transferred to a PVDF membrane (Millipore, Billerica, MA, USA). The membrane was blocked with 5% skim milk at room temperature for 1 h. After blocking, the membrane was incubated with the primary antibody overnight at 4°C, followed by incubation with the secondary antibody. Protein imaging was performed via an Amersham ImageQuant 800 Western blot imaging system (Cytiva, China), and quantitative analysis was performed using ImageJ software (National Institutes of Health, NIH, USA). All the antibodies used are listed in Table S1.

### 2.19 RT‐qPCR

Total RNA was extracted from lung tissue or cells using the Fastagen RNA Extraction Kit (Shanghai, China), and cDNA was synthesized via the Prime Script RT Master Mix Kit (TaKaRa, Tokyo, Japan) following the manufacturer's instructions. RT‐qPCR analysis was conducted on a Light Cycler instrument (Bio‐Rad, California, USA) with the FastStart Essential DNA Green Master Kit (Roche, Basel, Switzerland). The expression data were normalized to the mRNA level of GAPDH or the miRNA U6. Each sample was loaded in triplicate and analyzed using the 2^−ΔΔCt^ method. The sequences of the primers used are as follows:

Scd1 Forward: 5‐AGGCCTGTACGGGATCATACT‐3.

Scd1 Reverse: 5‐GCGCTGGTCATGTAGTAGAAAAT‐3.

mmu‐miR‐9‐5p Forward: 5‐AGCCTCTCTCCTCTTGGTTATCT‐3.

mmu‐miR‐9‐5p Reverse: 5‐TATGGTTGTTCTGCTCTCTGTGTC‐3.

### 2.20 Immunofluorescent (IF) Staining

Lung tissue sections were permeabilized with immunostaining permeabilization buffer for 5 min, then blocked with 5% BSA for 30 min at room temperature and incubated with Scd1 antibody diluted in 5% BSA overnight at 4°C. After washing away the primary antibody, the specimens were incubated with red fluorescent Alexa Fluor 594 rabbit anti‐mouse IgG (Invitrogen, Carlsbad, CA, USA) for 1 h at room temperature. Cell nuclei were stained with DAPI for 5 min. Changes in Scd1 were observed using confocal microscopy. All the antibodies used are listed in Table S1.

### 2.21 Statistical Analysis

All the statistical analyses were performed using GraphPad Prism 8.0 software (GraphPad Software, San Diego, CA, USA). The data are presented as the means ± standard deviations (SDs) from at least three independent experiments. Differences between the two groups were analyzed using Student's *t* test. For comparisons involving more than two groups, one‐way analysis of variance (ANOVA) followed by Bonferroni post hoc correction was used. A *p* value less than 0.05 was considered statistically significant.

## 3 Results

### 3.1 Brain‐Derived Exosomes Mediate TBI‐Induced ALI

**FIGURE 1:**
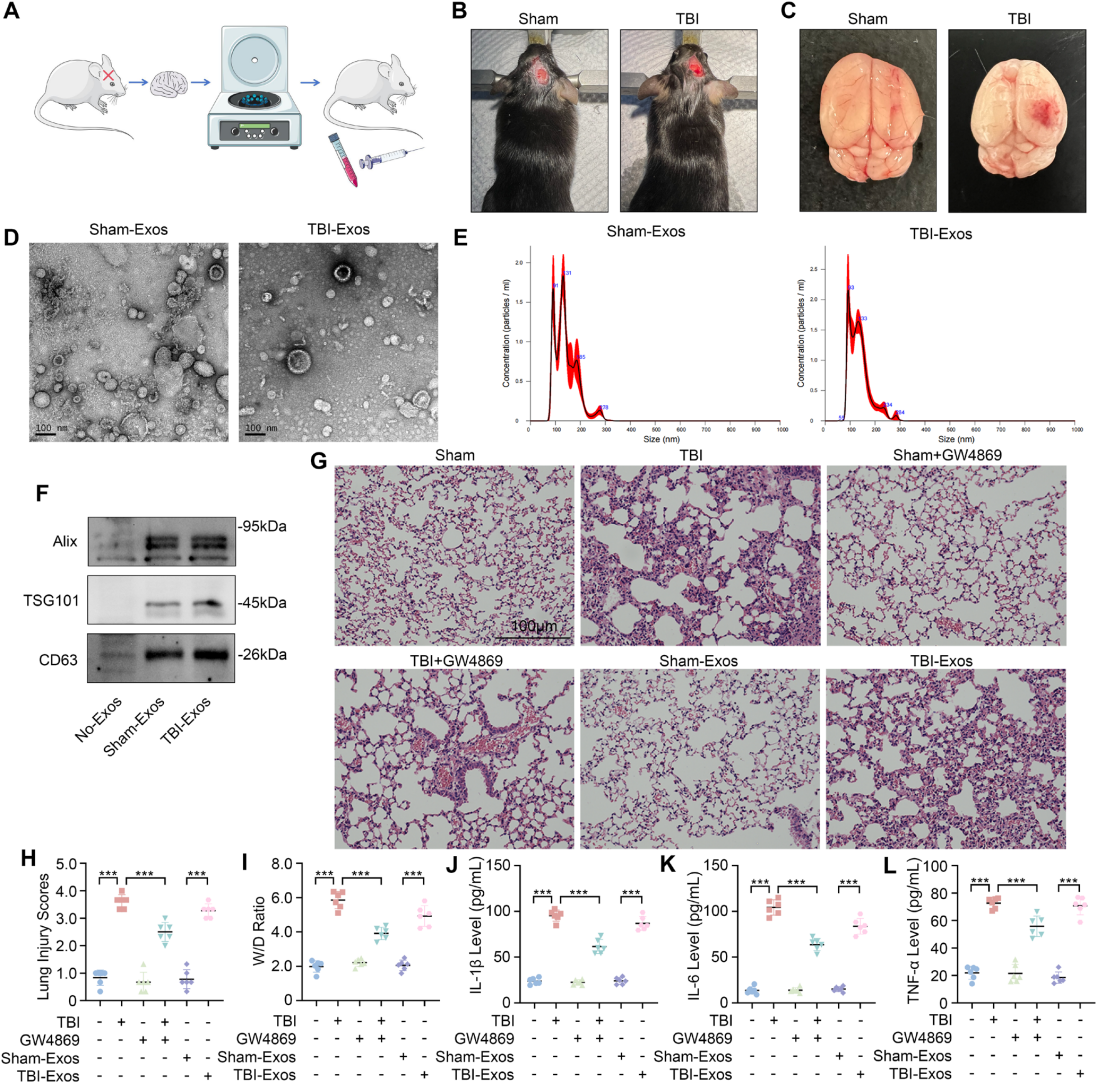
Brain‐derived exosomes cause TBI‐induced ALI. (A) Experimental flowchart. (B, C) Comparison of brain tissue damage after CCI. (D) Representative TEM images of the Sham‐Exos and TBI‐Exos. (E) Measurement of the particle size of the Sham‐Exos and TBI‐Exos via NTA. (F) Exosome representative markers detected via Western blot. (G, H) Lung injury score was evaluated via HE staining of the lung tissue of the mice (*n* = 6). (I) W/D weight ratio of mouse lung tissue (*n* = 6). (J–L) The levels of IL‐1β, IL‐6, and TNF‐α in mouse BALF (*n* = 6). *P < 0.05, **P < 0.01, ***P < 0.001, ns: not significant.

Exosomes were isolated from the brain tissues of the mice in both the Sham and TBI groups through differential centrifugation. Sham‐Exos and TBI‐Exos were subsequently injected into normal mice via the tail vein to evaluate their potential to induce lung damage (Figure 1A). The mice in the TBI group underwent CCI following craniotomy, resulting in evident cerebral congestion and tissue disruption. In contrast, the Sham group of mice only underwent craniotomy without CCI, maintaining intact brain tissue (Figure 1B,C).

Characterization of the exosomes via TEM and NTA revealed that both TBI‐Exos and Sham‐Exos displayed typical round‐ and cup‐shaped morphologies, with diameters predominantly between 100 and 200 nm (Figure 1D,E). Furthermore, the exosome‐specific markers Alix, TSG101, and CD63 were significantly expressed in both groups of exosomes, which further verified the purity and characteristics of the exosomes (Figure 1F).

The mice in the TBI group exhibited characteristics of ALI, including lung inflammation, hemorrhage, necrosis, atelectasis, pulmonary edema, and hyaline membrane formation. Interestingly, the severity of lung injury in TBI mice was significantly mitigated following treatment with GW4869. In addition, mice injected with TBI‐Exos also exhibited characteristics of ALI, whereas mice injected with Sham‐Exos did not (Figure 1G).

The lung injury score, W/D ratio, and levels of IL‐1β, IL‐6, and TNF‐α in the BALF (Figure 1H–L) revealed that treatment with GW4869 significantly reduced the extent of lung injury in TBI mice and TBI‐Exo‐induced ALI in mice, indicating that brain‐derived exosomes mediated TBI‐induced ALI.

### 3.2 TBI Causes Ferroptosis in Lung Tissue

**FIGURE 2:**
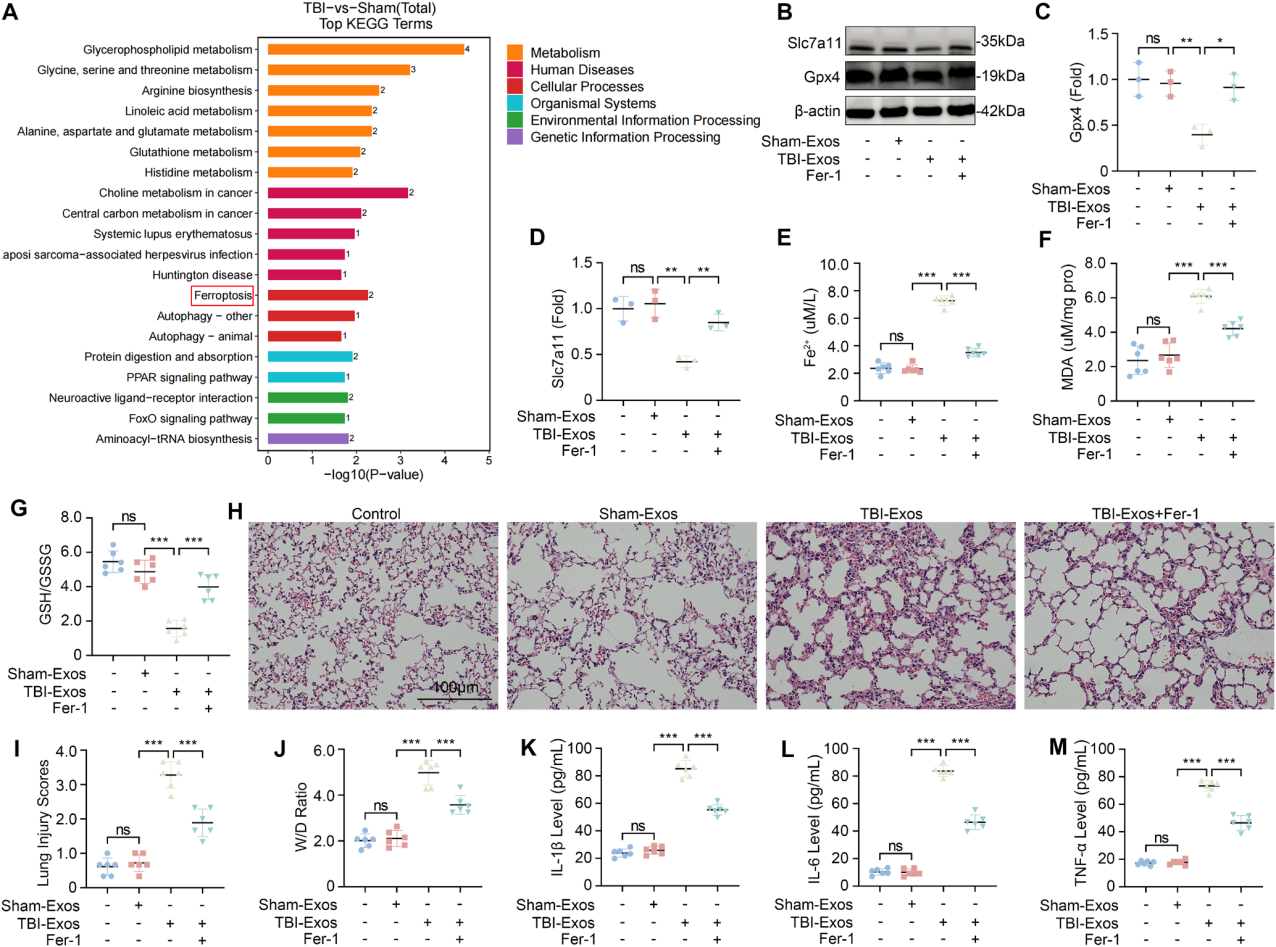
TBI leads to ferroptosis. (A) LC‐MS nontargeted metabolomics analysis of metabolic differences in lung tissues between the Sham group and the TBI group. (B–D) Western blotting was used to detect the expression levels of Slc7a11 and Gpx4 in mouse lung tissues after the injection of Sham‐Exos, TBI‐Exos, or TBI‐Exos+Fer‐1 (*n* = 3). (E) The concentration of Fe^2+^ in mouse lung tissues (*n* = 6). (F) Concentrations of MDA in mouse lung tissues (*n* = 6). (G) The ratio of GSH/GSSG in mouse lung tissues (*n* = 6). (H, I) Lung injury score was evaluated via HE staining of the lung tissue of the mice (*n* = 6). (J) W/D weight ratio of mouse lung tissue (*n* = 6). (K, L, M) Levels of IL‐1β, IL‐6, and TNF‐α in mouse BALF (*n* = 6). *P < 0.05, **P < 0.01, ***P < 0.001, ns: not significant.

To explore the pathological mechanism of TBI‐induced ALI, we compared the metabolic changes in the lung tissue of the mice in the Sham and TBI groups through LC‐MS nontargeted metabolic analysis. Our findings revealed increased enrichment of pathways associated with ferroptosis in the lung tissues of the TBI group relative to those of the Sham group (Figure 2A). The inhibition of glutathione peroxidase 4 (Gpx4) is considered to be a key factor in inducing ferroptosis [25]. Additionally, the inhibition of solute carrier family 7 member 11 (Slc7a11) has been shown to impair cystine uptake, leading to the inactivation of cystine‐dependent glutathione peroxidase and the promotion of ferroptosis [26]. We assessed the expression levels of Gpx4 and Slc7a11 in the lung tissues of mice and observed a significant reduction in these proteins in mice injected with TBI‐Exos compared with those in mice injected with Sham‐Exos. However, treatment with the ferroptosis inhibitor Fer‐1 restored the expression levels of Gpx4 and Slc7a11 (Figure 2B–D).

The accumulation of Fe^2+^ and the lipid peroxidation product MDA are hallmarks of ferroptosis [27, 28]. Our assays demonstrated that the injection of TBI‐Exos led to a significant increase in both the Fe^2+^ and MDA concentrations, which were mitigated by Fer‐1 treatment (Figure 2E,F). In addition, we also examined the GSH/GSSG ratio, which is an important indicator of oxidative stress and ferroptosis levels [29]. The results revealed that the GSH/GSSG ratio in the lung tissue of the mice was significantly reduced after the injection of TBI‐Exos, whereas Fer‐1 treatment increased this ratio (Figure 2G). Collectively, these results suggest that exosomes released after TBI can facilitate the progression of ferroptosis and that Fer‐1 can inhibit this process.

To further verify the role of ferroptosis in TBI‐induced ALI, we evaluated the degree of lung injury in the mice in each group. The results showed that Fer‐1 treatment could alleviate ALI caused by TBI‐Exos (Figure 2H–M). Therefore, we conclude that ferroptosis plays a key role in the pathological process of TBI‐induced ALI.

### 3.3 miR‐9‐5p Is a Potential Functional Component of Exosomes

**FIGURE 3:**
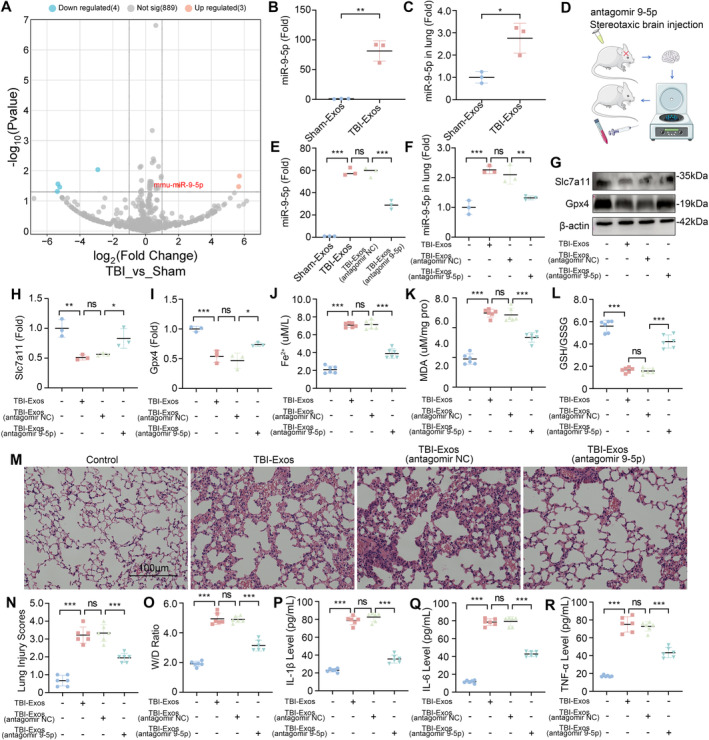
miR‐9‐5p is a potential functional component of exosomes. (A) Comparison of DE‐miRNAs in the lung tissues of Sham group and TBI group. (B) RT‐qPCR analysis of the miR‐9‐5p expression level in Sham‐Exos and TBI‐Exos (*n* = 3). (C) RT‐qPCR analysis of the miR‐9‐5p expression level in lung tissues after the injection of the Sham‐Exos or TBI‐Exos (*n* = 3). (D) antagomir 9‐5p was injected into the brains of mice after CCI, and exosomes were extracted and then injected into normal mice. (E) RT‐qPCR analysis of the miR‐9‐5p expression level in Sham‐Exos, TBI‐Exos, TBI‐Exos (antagomir NC), and TBI‐Exos (antagomir 9‐5p) (*n* = 3). (F) RT‐qPCR analysis of the miR‐9‐5p expression level in the lung tissues of mice after the injection of TBI‐Exos, TBI‐Exos (antagomir NC), and TBI‐Exos (antagomir 9‐5p) (*n* = 3). (G, H, I) Western blotting was used to detect the expression levels of Slc7a11 and Gpx4 in mouse lung tissues (*n* = 3). (J) Concentration of Fe^2+^ in mouse lung tissues (*n* = 6). (K) The concentration of MDA in mouse lung tissues (*n* = 6). (L) The ratio of GSH/GSSG in mouse lung tissues (*n* = 6). (M, N) Lung injury score was evaluated via HE staining of the lung tissue of the mice (*n* = 6). (O) W/D weight ratio of mouse lung tissue (*n* = 6). (P, O, R) Levels of IL‐1β, IL‐6, and TNF‐α in mouse BALF (*n* = 6). *P < 0.05, **P < 0.01, ***P < 0.001, ns: not significant.

To elucidate the mechanisms by which exosomes contribute to ferroptosis in lung tissue following TBI, we conducted miRNA microarray analysis on the lung tissue of mice in the Sham and TBI groups. A total of seven differentially expressed miRNAs (DE‐miRNAs) were identified, three of which were upregulated and four of which were downregulated (Figure 3A). Among the upregulated miRNAs, the upregulation of miR‐9‐5p attracted our interest [30]. Further validation through RT‐qPCR confirmed that the miR‐9‐5p level was significantly elevated in the TBI‐Exos group compared with the Sham‐Exos group (Figure 3B). This trend was mirrored in the lung tissues of the mice, where the miR‐9‐5p levels were also significantly greater following the injection of the TBI‐Exos than those following the Sham‐Exos (Figure 3C).

To further explore the function of miR‐9‐5p in brain‐derived exosomes, we performed brain‐targeted injections of antagomir 9‐5p or antagomir NC in mice subjected to CCI. Subsequently, we re‐injected the exosomes extracted from the mouse brain tissues back into the mice (Figure 3D). The results showed that the activity of miR‐9‐5p in both the extracted exosomes and the lung tissues of mice after exosome injection was significantly inhibited by antagomir 9‐5p (Figure 3E,F). We found that the expression of Slc7a11 and Gpx4 in the lung tissue of mice injected with TBI‐Exos (antagomir 9‐5p) was significantly greater than that in the lung tissue of mice injected with TBI‐Exos or TBI‐Exos (antagomir NC) (Figure 3G–I). In addition, the measurement of Fe^2+^, MDA and the GSH/GSSG ratio in the lung tissue revealed that the TBI‐Exos and the TBI‐Exos (antagomir NC) induced ferroptosis in the lung tissue, whereas the ferroptotic response induced by the TBI‐Exos (antagomir 9‐5p) was markedly diminished (Figure 3J–L).

While investigating the correlation between miR‐9‐5p and lung injury, HE staining revealed that, compared with that in the mice injected with the TBI‐Exos or the TBI‐Exos (antagomir NC), the degree of lung damage in the mice injected with the TBI‐Exos antagomir‐9‐5p was lower (Figure 3M). Moreover, the mice injected with the TBI‐Exos (antagomir 9‐5p) had lower pulmonary edema and less inflammatory factor release (Figure 3N–R). These observations suggest that miR‐9‐5p is a key functional component of brain‐derived exosomes that mediate ferroptosis in lung tissue following TBI.

### 3.4 Brain‐Derived Exosome Delivery of miR‐9‐5p Induces Ferroptosis in MLE‐12 Cells After TBI

**FIGURE 4:**
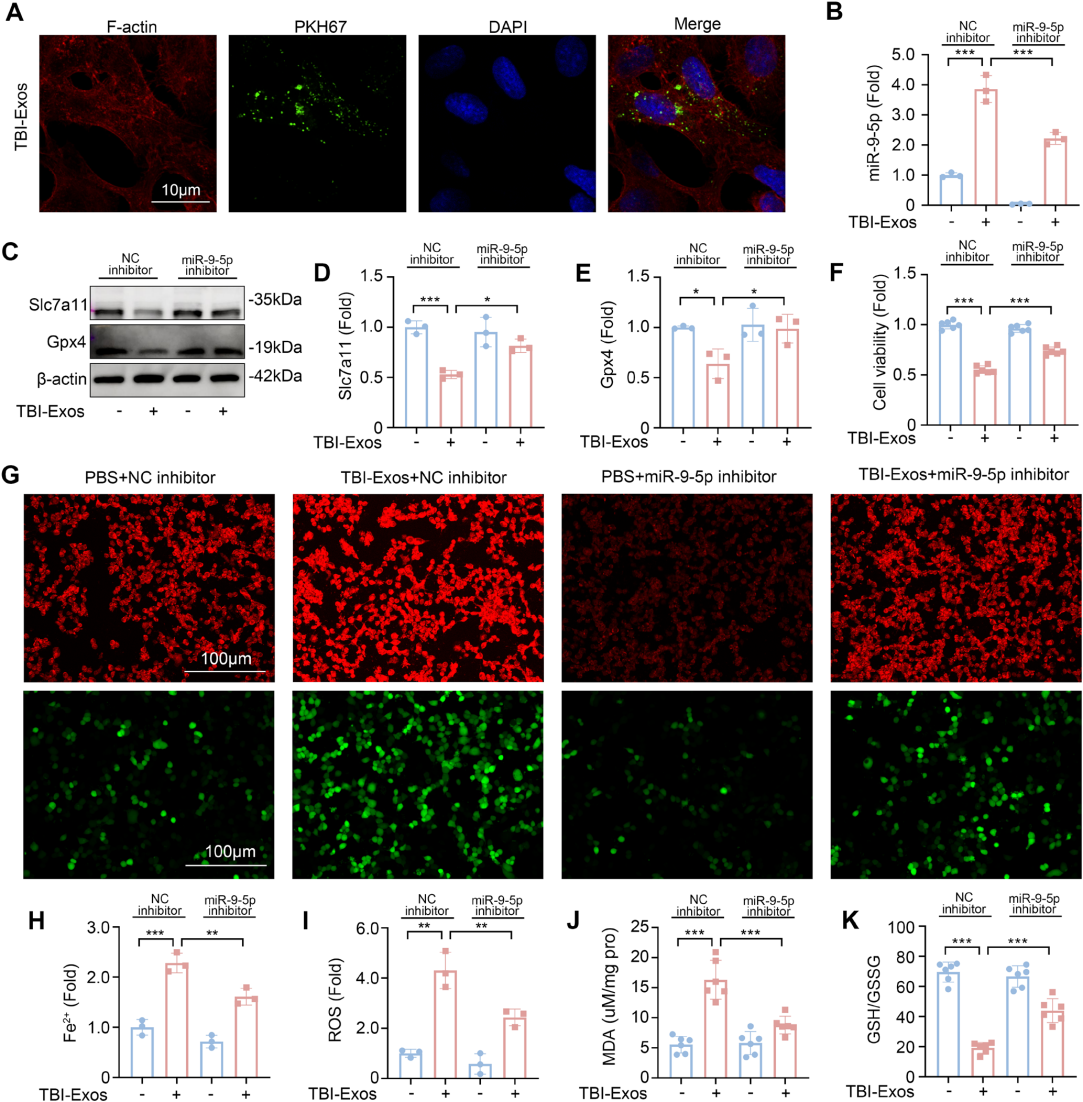
Brain‐derived exosomes and miR‐9‐5p induce ferroptosis in MLE‐12 cells after TBI. (A) Confocal image of MLE‐12 cells incubated with PKH67‐labeled exosomes. (B) The expression level of miR‐9‐5p in MLE‐12 cells after coincubation with TBI‐Exos or transfection with the miR‐9‐5p inhibitor was detected by RT‐qPCR (*n* = 3). (C–E) Western blotting was used to detect the expression levels of Slc7a11 and Gpx4 in MLE‐12 cells (*n* = 3). (F) Detection of cell viability via a CCK8 kit (*n* = 6). (G–I) confocal images of MLE‐12 cells coincubated with the Fe^2+^ fluorescent probe (red dye)/ROS fluorescent probe (green dye) (*n* = 3). (J) The concentration of MDA in MLE‐12 cells (*n* = 6). (K) The ratio of GSH/GSSG in MLE‐12 cells (*n* = 6). *P < 0.05, **P < 0.01, ***P < 0.001, ns: not significant.

Disruption of the blood–air barrier (BAB) is a key pathological characteristic of ALI, with alveolar epithelial cells being an integral component of the BAB [31, 32]. Using confocal microscopy, we observed the uptake of fluorescently labeled exosomes within the cytoplasm of MLE‐12 cells, thereby confirming the effective internalization of TBI‐Exos by these cells (Figure 4A). After coculture, the expression level of miR‐9‐5p in MLE‐12 cells increased significantly, indicating that brain‐derived exosomes delivered miR‐9‐5p to MLE‐12 cells. We then studied the function of miR‐9‐5p by transfecting the cells with a miR‐9‐5p inhibitor (Figure 4B). Our findings revealed that coculture with TBI‐Exos led to a reduction in the expression of Slc7a11 and Gpx4 in MLE‐12 cells, an effect that was mitigated by the miR‐9‐5p inhibitor (Figure 4C–E).

The results of the analysis of cell activity revealed that the miR‐9‐5p inhibitor inhibited the decrease in cell activity caused by the TBI‐Exos (Figure 4F). Given that the accumulation of Fe^2+^ and reactive ROS is pivotal in the induction of ferroptosis [33, 34], we utilized fluorescent probes to visualize these species within cells. Fluorescence microscopy revealed that TBI‐Exos significantly increased the intracellular levels of Fe^2+^ and ROS in MLE‐12 cells, whereas the miR‐9‐5p inhibitor effectively suppressed this accumulation (Figure 4G,H). In addition, the MDA content (Figure 4J) and GSH/GSSG ratio in MLE‐12 cells (Figure 4K) further confirmed that brain‐derived exosomes induced ferroptosis in MLE‐12 cells after TBI through miR‐9‐5p.

### 3.5 miR‐9‐5p Mediates Ferroptosis via Scd1

**FIGURE 5:**
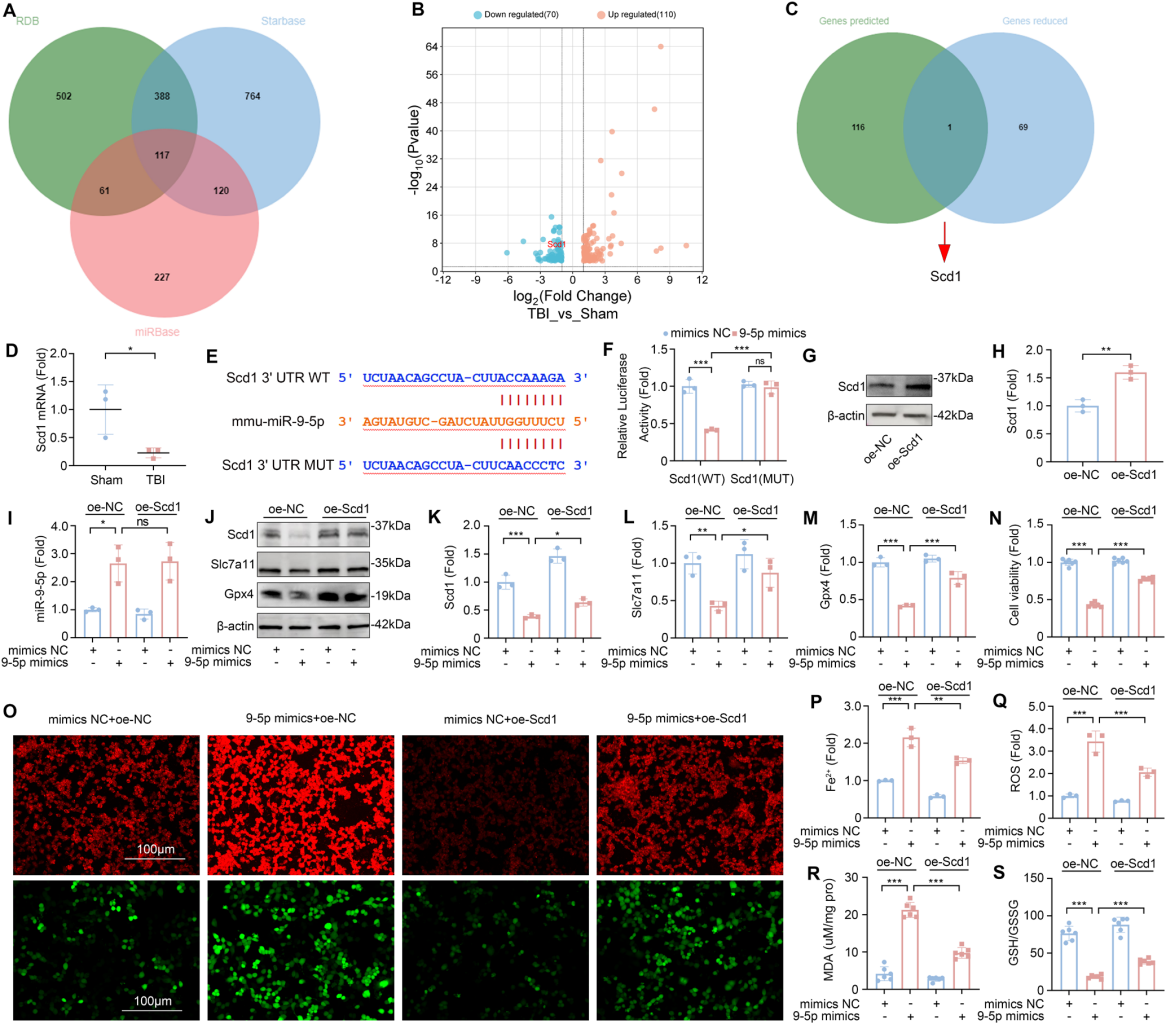
miR‐9‐5p mediates ferroptosis via Scd1. (A) 117 miR‐9‐5p target genes were predicted via the miRBase, StarBase, and RDB platforms. (B) Volcano plot of DEGs between the lung tissues of the Sham and TBI groups. (C) Cross‐analysis of predicted target genes and downregulated genes via mRNA sequencing. (D) Scd1 mRNA expression levels in the lung tissue of the Sham group and TBI group determined via RT‐qPCR (*n* = 3). (E) Predicted binding site between miR‐9‐5p and Scd1. (F) Dual‐luciferase reporter gene experiments confirmed the relationship between miR‐9‐5p and Scd1 (*n* = 3). (G, H) Efficiency verification of overexpression of Scd1 (*n* = 3). (I) The expression level of miR‐9‐5p in MLE‐12 cells after transfection with Scd1 or with miR‐9‐5p mimics as detected via RT‐qPCR (*n* = 3). (J–M) Western blotting was used to detect the expression levels of Scd1, Slc7a11, and Gpx4 in MLE‐12 cells (*n* = 3). (N) Detection of cell viability by CCK8 kit (*n* = 6). (O–Q) Confocal images of MLE‐12 cells coincubated with the Fe^2+^ fluorescent probe (red dye)/ROS fluorescent probe (green dye) (*n* = 3). (R) The concentration of MDA in MLE‐12 cells (*n* = 6). (S) The ratio of GSH/GSSG in MLE‐12 cells (*n* = 6). *P < 0.05, **P < 0.01, ***P < 0.001, ns: not significant.

To further explore the mechanism of miR‐9‐5p in TBI‐induced ALI, we investigated potential downstream target genes of miR‐9‐5p. Utilizing three bioinformatics databases—miRBase, starBase, and RDB, we identified 117 candidate target genes (Figure 5A). Concurrently, we conducted a comparative mRNA sequencing analysis of lung tissues from the mice in the TBI and Sham groups. This analysis revealed that 110 genes were significantly upregulated, whereas 70 genes were downregulated in the TBI group (Figure 5B). By intersecting the differentially downregulated genes with our predicted targets, we identified Scd1 as a potential target of miR‐9‐5p (Figure 5C). Furthermore, previous research suggests that the suppression of Scd1 expression can induce ferroptosis [35]. This notion was corroborated by our subsequent validation via RT‐qPCR, which revealed a significant reduction in the mRNA levels of Scd1 in the lung tissues of the TBI group compared with those in the Sham group (Figure 5D).

To confirm the regulatory interaction between miR‐9‐5p and Scd1, we conducted a dual‐luciferase reporter assay. The results demonstrated that the overexpression of miR‐9‐5p significantly diminished the luciferase activity of the Scd1‐3′UTR‐WT reporter gene (Figure 5E,F) but had no significant effect on the activity of the Scd1‐3′UTR‐MUT reporter gene. These findings establish that miR‐9‐5p specifically targets and downregulates the expression of Scd1, thereby providing a mechanistic link between miR‐9‐5p and its downstream effector in the context of TBI‐induced ALI.

To further investigate the role and mechanism of Scd1 in miR‐9‐5p‐mediated TBI‐induced ALI, we transfected MLE‐12 cells with miR‐9‐5p mimics and Scd1 plasmids and verified the effectiveness of the transfection (Figure 5G–I). Compared with those in the control group, the expression of Slc7a11 and Gpx4 decreased after the cells were transfected with the miR‐9‐5p mimics. When the miR‐9‐5p mimics and Scd1 plasmid were simultaneously transfected, the expression levels of Slc7a11 and Gpx4 were upregulated in MLE‐12 cells (Figure 5J–M). The results of the CCK8 toxicity test also revealed that the overexpression of Scd1 inhibited the decrease in cell activity caused by the miR‐9‐5p mimics (Figure 5N).

To evaluate the degree of ferroptosis in MLE‐12 cells more intuitively, we incubated cells subjected to different transfection treatments with an Fe^2+^ fluorescent probe or an ROS fluorescent probe. A fluorescence microscope revealed that the fluorescence intensity of Fe^2+^ and ROS in MLE‐12 cells after cotransfection was significantly lower than that in cells transfected with the miR‐9‐5p mimics alone (Figure 5O–Q). In addition, the results of the kit test also revealed that the overexpression of Scd1 inhibited the increase in the intracellular MDA concentration and the decrease in the GSH/GSSG ratio caused by the miR‐9‐5p mimics (Figure 5R,S). These results indicate that miR‐9‐5p in TBI‐Exos induces ferroptosis by targeting the downregulation of Scd1 in MLE‐12 cells.

### 3.6 Antagomir 9‐5p Treatment Alleviates TBI‐Induced ALI

**FIGURE 6:**
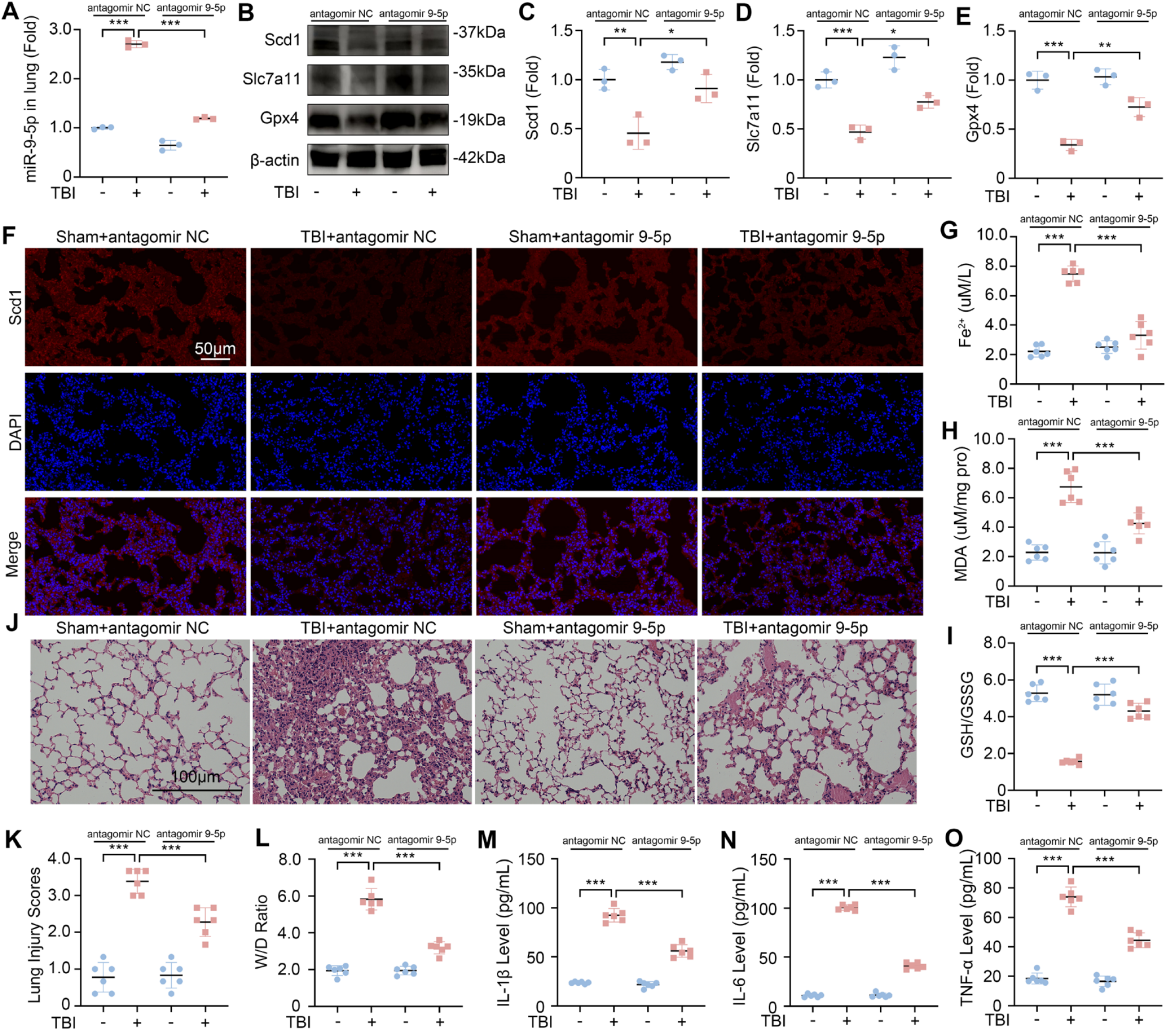
Antagomir 9‐5p treatment alleviates TBI‐induced ALI. (A) RT‐qPCR analysis of the miR‐9‐5p expression level in the lung tissues of the mice in the Sham group, TBI group, antagomir 9‐5p treatment group, and antagomir 9‐5p treatment group after TBI (*n* = 3). (B–E) Western blot analysis of the expression levels of Scd1, Slc7a11, and Gpx4 in mouse lung tissues (*n* = 3). (F) Representative IF staining of Scd1 in mouse lung tissue. (G) Concentrations of Fe^2+^ in mouse lung tissues (*n* = 6). (H) The concentration of MDA in mouse lung tissues (*n* = 6). (I) The ratio of GSH/GSSG in mouse lung tissues (*n* = 6). (J, K) Lung injury score was evaluated via HE staining of the lung tissue of the mice (*n* = 6). (L) W/D weight ratio of mouse lung tissue (*n* = 6). (M–O) Levels of IL‐1β, IL‐6, and TNF‐α in mouse BALF (*n* = 6). *P < 0.05, **P < 0.01, ***P < 0.001, ns: not significant.

To verify the therapeutic potential of antagomir 9‐5p against TBI‐induced ALI, we administered antagomir 9‐5p via nasal instillation after the mice underwent CCI. After treatment with antagomir 9‐5p, the level of miR‐9‐5p in the lung tissue of mice was significantly reduced (Figure 6A). Compared with those in the Sham group, the expression levels of Scd1, Slc7a11, and Gpx4 in the lung tissue of the mice in the TBI group were significantly lower. After treatment with antagomir 9‐5p, the expression levels of these three markers increased (Figure 6B–E). The outcomes of IF staining on lung tissue revealed that the reduction in Scd1 expression induced by TBI was markedly mitigated following the inhibition of miR‐9‐5p, as compared to the levels observed prior to treatment (Figure 6F). In addition, the detection of Fe^2+^, MDA, and the GSH/GSSG ratio in lung tissue revealed that antagomir 9‐5p treatment significantly inhibited TBI‐induced ferroptosis (Figure 6G–I).

We evaluated the degree of ALI in the lung tissue of the Sham group, TBI, antagomir 9‐5p group, and TBI + antagomir 9‐5p group. HE staining results showed that compared with the TBI group, the inflammatory cell infiltration and lung interstitial congestion in the lung tissue of the TBI + antagomir 9‐5p group were significantly lower (Figure 6J). Similarly, after treatment with antagomir 9‐5p, the lung injury score and W/D ratio of the mice after TBI were significantly reduced, and the levels of IL‐1β, IL‐6, and TNF‐α in the BALF were also significantly reduced (Figure 6K–O). These results show that the administration of antagomir 9‐5p is a promising therapeutic approach for inhibiting ferroptosis and alleviating the severity of TBI‐induced ALI.

## 4 Discussion

Our study reveals the key role of miR‐9‐5p in brain‐derived exosomes after TBI, which induced ferroptosis in lung epithelial cells by targeting Scd1, thus playing an important role in the pathological process of TBI‐induced ALI. In addition, we also found that treatment with antagomir 9‐5p effectively inhibited ferroptosis after TBI, thereby ameliorating TBI‐induced ALI.

In previous studies, the mechanisms of exosome‐related TBI‐induced ALI were summarized as coagulation dysfunction, the inflammatory response, and destruction of the BAB [36]. The majority of these studies underscore the critical involvement of inflammasomes in brain‐derived exosomes in the inflammatory response associated with TBI‐induced ALI [37]. However, other mechanisms of the exosome‐mediated inflammatory response after TBI‐induced ALI have rarely been studied. By employing LC‐MS for nontargeted metabolic profiling, our study revealed increased metabolic enrichment of ferroptosis in lung tissues following TBI.

Injection of TBI‐Exos via the tail vein can lead to a decrease in the expression of Gpx4 and Slc7a11 in the lung tissue of mice, accompanied by an increase in multiple ferroptosis markers and inflammatory indicators, whereas Fer‐1 can inhibit this trend. Therefore, we hypothesize that exosomes originating from the brain after TBI not only trigger ferroptosis in lung tissues but also facilitate the development of ALI and that there is a close connection between ferroptosis and ALI.

The analysis of the miRNA microarray data from the lung tissues of the mice in the TBI and the Sham groups highlights the intriguing role of miR‐9‐5p. Previous studies have shown that miR‐9‐5p plays an important role in promoting ferroptosis in sepsis‐related encephalopathy [30]. To explore the regulatory role of miR‐9‐5p in ferroptosis in TBI‐induced ALI, we injected antagomir 9‐5p into the brains of TBI model mice to antagonize miR‐9‐5p in exosomes. Our findings revealed that, compared with those injected solely with TBI‐Exos, the mice treated with TBI‐Exos (antagomir 9‐5p) exhibited a reduced extent of ferroptosis and lung inflammation. This animal experiment demonstrated that miR‐9‐5p is a key molecule for TBI‐Exos‐induced ferroptosis and ALI.

The alveolar capillaries in the BAB are composed mainly of type I and type II alveolar epithelial cells and endothelial cells, and BAB destruction is a key pathological feature of ALI [31, 32]. Previous research suggests that specific cargo within TBI‐Exos may directly damage alveolar capillary endothelial cells, resulting in BAB dysfunction during TBI‐induced ALI [38]. However, the precise role of epithelial cells in this pathological cascade remains to be fully delineated. Using the MLE‐12 murine lung epithelial cell line, we investigated the impact of TBI‐Exos on lung epithelial cells. Our study demonstrated that TBI‐Exos were effectively internalized by MLE‐12 cells, leading to a decrease in the expression of the key intracellular ferroptosis markers Gpx4 and Slc7a11, accompanied by the accumulation of intracellular Fe^2+^ and ROS, indicating ferroptosis of lung epithelial cells. In addition, by using a miR‐9‐5p inhibitor to inhibit miR‐9‐5p in TBI‐Exos cocultured with cells, we observed a rebound in the expression of intracellular Gpx4 and Slc7a11, a decrease in the accumulation of Fe^2+^ and ROS, and a decrease in the concentration of ferroptosis markers such as MDA. The results of these cell experiments revealed that during TBI‐induced ALI, miR‐9‐5p triggered ferroptosis in MLE‐12 cells through TBI‐Exos, leading to the destruction of BAB and the occurrence of ALI.

Through comprehensive analysis via multiple bioinformatics databases and corroborating these predictions with our mRNA sequencing data, we identified Scd1 as a potential target gene of miR‐9‐5p. Scd1, a crucial lipase enzyme localized in the endoplasmic reticulum, is known to play a significant role in the regulation of ferroptosis. It has been shown to exert a negative regulatory effect on ferroptosis in various cancers [39, 40, 41, 42]. However, its role in the context of ALI has not been extensively investigated. Our initial findings confirmed that miR‐9‐5p can directly target Scd1 and that the miR‐9‐5p mimics in MLE‐12 cells resulted in a reduction in Scd1 expression and an increase in ferroptosis. Intriguingly, cotransfection of miR‐9‐5p mimics with Scd1 overexpression plasmids in MLE‐12 cells led to a decrease in the degree of intracellular ferroptosis. These results not only substantiate the positive regulatory effect of miR‐9‐5p on ferroptosis but also suggest that the inhibition of Scd1 may promote ferroptosis in ALI, whereas Scd1 overexpression may mitigate this process.

In our study, analysis of lung tissues from TBI model mice revealed that decreased Scd1 expression was accompanied by increased levels of ferroptosis and lung injury, which aligns with our previous cellular experimental observations. Moreover, the administration of antagomir 9‐5p via nasal instillation in TBI mice resulted in the restoration of Scd1 expression in lung tissues, along with a reduction in both ferroptosis and ALI levels. These findings highlight the therapeutic potential of miR‐9‐5p in targeting the Scd1‐mediated ferroptosis axis in TBI‐induced ALI and provide a new perspective for the study of Scd1‐related ferroptosis, which may not be limited to Scd1 overexpression [39, 40].

Together, our in vitro and in vivo experimental results revealed that miR‐9‐5p in TBI‐Exos induces ferroptosis by targeting and downregulating Scd1 in lung epithelial cells, further triggering an enhanced inflammatory response in lung tissues and destruction of BAB, ultimately leading to TBI‐induced ALI.

Although our study provides a detailed explanation of the mechanism of miRNA‐mediated ferroptosis in TBI‐induced ALI, it also has limitations. For example, we have not yet been able to determine the exact source of TBI‐Exos, nor have we explored in detail the specific mechanisms of action of different components of TBI‐Exos on ALI. In this study, even after antagonizing miR‐9‐5p in TBI‐Exos, ferroptosis was not completely inhibited. This phenomenon may be related to the inhibitory effects of the miR‐9‐5p inhibitor and antagomir 9‐5p or to the regulatory effects of other components of TBI‐Exos on ferroptosis.

## 5 Conclusion

This study demonstrates that brain‐derived exosomes mediate ferroptosis in TBI‐induced ALI by targeting and regulating Scd1 through miR‐9‐5p. These findings may provide a new perspective for understanding the complex interaction between TBI and ALI and highlight the possibility that miR‐9‐5p may be a potential target in the development of new therapeutic strategies.

## Author Contributions

**Yi Zhang:** conceptualization, Methodology, Investigation, Formal analysis, Writing – original draft, Writing – review and editing. **Chang Sun:** conceptualization, Investigation, Writing – original draft, Writing – review and editing. **Bailun Wang:** investigation, Data curation. **Angran Gu:** investigation, Data curation. **Ziyi Zhou:** software, Visualization. **Changping Gu:** conceptualization, Resources, Supervision, Writing – review and editing.

## Conflicts of Interest

The authors declare no conflicts of interest.

## Supporting information


Table S1.

## Data Availability

The datasets used and/or analyzed during the current study are available from the corresponding author upon reasonable request.
