# Supplementary material for: Brain‐Derived Exosomal miR‐9‐5p Induces Ferroptosis in Traumatic Brain Injury‐Induced Acute Lung Injury by Targeting Scd1
Source: CNS Neurosci Ther. 2024 Dec 26;30(12):e70189. doi: 10.1111/cns.70189 (PMC11669946; doi:10.1111/cns.70189)
Supplement: Supplementary file 1 — Table S1. [file CNS-30-e70189-s001.docx]

**Supplementary Table S1 Information of antibody**

| Antibody name | Company | Catalog number | Source | Reactive | Dilution |
| --- | --- | --- | --- | --- | --- |
| CD63 | Santa Cruz | sc-5275 | Mouse | M, H, R | WB 1:200 |
| Alix | Santa Cruz | sc-53540 | Mouse | M, H, R | WB 1:200 |
| TSG101 | Santa Cruz | sc-7964 | Mouse | M, H, R | WB 1:200 |
| Beta Actin | Proteintech | 66009-1-Ig | Mouse | M, H, R | WB 1:20000 |
| Gpx4 | Proteintech | 67763-1-Ig | Mouse | M, H, R | WB 1:1000 |
| xCT/Slc7a11 | CST | #98051 | Rabbit | M | WB 1:1000 |
| Scd1 | CST | #2438S | Rabbit | M, H | WB 1:1000 |
| Scd1 | CST | #2438S | Rabbit | M, H | IF 1:100 |
